# Malignant Recurrence of Benign Odontogenic Tumors (A Single Center Cross-Sectional Study)

**DOI:** 10.1007/s12105-024-01676-9

**Published:** 2024-08-13

**Authors:** Manar Abdul-Waniss Mohammed Abdul-Aziz, Asmaa Emad El-Din Mohammed Rashad, Heba Ahmed Saleh

**Affiliations:** Oral and Maxillofacial Pathology, Faculty of Dentistry, Cairo University, 11 el Saraya Street, Manyal, Cairo, Egypt

**Keywords:** Recurrent odontogenic tumors, Odontogenic malignancy, Malignant transformation, Odontogenic neoplasms, Epidemiology

## Abstract

**Background:**

Despite their rarity, malignant odontogenic tumors (MOT) represent an important group of oral lesions characterized by their variable clinical presentations and sometimes unexpected biological behavior.

**Objectives:**

The purpose of this retrospective cross-sectional study was to evaluate the number, types, and frequency of MOT and to investigate the relative rate of malignant transformation in recurrent odontogenic tumors (OT).

**Methodology:**

The records of patients diagnosed with OT in the hospital of the Faculty of Dentistry, Cairo University, were reviewed over 10 years (2013-2022). The OT were investigated for frequency, age, gender, site, and recurrence. The data were recorded and then analyzed using SPSS software version 25.

**Results:**

Among 5543 oral excisions, 357 cases of them were OT, including 336 benign (94.1%) and 21 malignant neoplasms (5.9%). Among the odontogenic malignancies, 18 lesions (85.7%) appeared de novo, and 3 lesions (14.3%) developed as recurrent of previously classified benign tumors. A high incidence was observed in the middle and old age groups (90.4%) with a median age being 42. Slight male predilection (1.3:1) was noticed. The mandible was the highly affected site but all recurrent cases were diagnosed in the maxilla as ghost cell odontogenic carcinoma (*n* = 2, 66.6%) and primary intraosseous carcinoma (*n* = 1, 33.3%).

**Conclusion:**

Retrospective analysis of the relative frequency of MOT and the documentation of the unusual recurrence of benign OT as a malignancy enhances our understanding of OT behavior and the need for appropriate therapy and clinical follow-up.

## Introduction

Odontogenic tumors (OT) comprise a large group of heterogeneous lesions arising from odontogenic (epithelial and/or mesenchymal) tissues of the tooth-forming apparatus [1]. Mostly, OT develop either de novo or within pre-existing odontogenic cysts. They possess different clinical behavior and histopathological features ranging from non-neoplastic lesions, hamartomas, benign neoplasia, and locally infiltrative and malignant neoplasia. Malignancies account for < 1% of all neoplastic lesions, except for metastatic odontogenic lesions, within the maxillofacial bones or in the soft tissue overlying tooth-bearing areas [2].

Attributable to their origin, OT usually show epithelial-mesenchymal interactions and subsequently inductive changes. These interactions were reflected in the World Health Organization’s most recent classification of OT, which categorizes them into epithelial, mesenchymal, or mixed lesions, depending on which component of the odontogenic apparatus gives rise to the tumor [3].

Malignant odontogenic tumors (MOT) are a rare entity that comprises carcinomas, sarcomas, or carcinosarcoma [3]. Certain benign odontogenic lesions are well-known for their high recurrence rate including odontogenic keratocyst (OKC) and ameloblastoma (AB) [4]. However, the probability of malignant transformation of these lesions when recurred is still unclear.

This retrospective cross-sectional study aimed to assess the number, types, and frequency of MOT diagnosed de novo or as a recurrence from previous benign OT over 10 years (2013–2022), at the Dental Educational Hospital of the Faculty of Dentistry, Cairo University, based on the classification of WHO of Head and Neck Tumors in 2022, as this information has not been reported previously in Egypt. The results are compared with the available global reports [5].

## Methodology

This study proposal was revised and approved by the Research Ethics Committee, Faculty of Dentistry, Cairo University (no.13 2 22). All methods were permitted to be done in accordance with the relevant guidelines and regulations of the Research Ethics Committee. Patients’ names included in the histopathological reports were kept confidential and were not utilized in this study. Given the retrospective, anonymized nature of the study, informed consent was waived by our internal review board.

Retrospective archival records of the Oral and Maxillofacial Pathology Department at the Educational Dental Hospital, Faculty of Dentistry, Cairo University, were examined for the cases that were diagnosed as MOT between 2013 and 2022. Inclusion criteria involved all MOT diagnosed in the selected period with complete data and clear confirmed diagnosis.

Clinical, radiological, and histopathological reports of the included cases were checked manually and electronically. Data regarding the patients’ age, sex, and anatomical location of OT was extracted. The history of each case was also studied to find the recurrent cases and the time interval between primary and recurrent lesions was recorded.

For all cases, Hematoxylin and Eosin stained tissue sections of MOT were re-examined under light microscopy by three experienced pathologists. In recurrent cases, the initial lesions were also retrieved and re-examined. The final diagnosis was confirmed or modified based on the WHO classification of Head and Neck Tumors in 2022 [5].

Descriptive statistical analysis was performed with all collected data using SPSS software, (version 25; SPSS, Inc, Chicago, IL). The graph was used to describe the distribution pattern of MOT according to the year of diagnosis. Tables were used to allocate the distribution of MOT concerning the different age groups, sex, and site. Recurrent cases of MOT were tabulated according to age, sex, site, clinical and radiographic features, the primary tumors, and the time interval between primary and recurrent tumors.

## Results

### Frequency and Distribution of MOT During the Studied Period

**Fig. 1:**
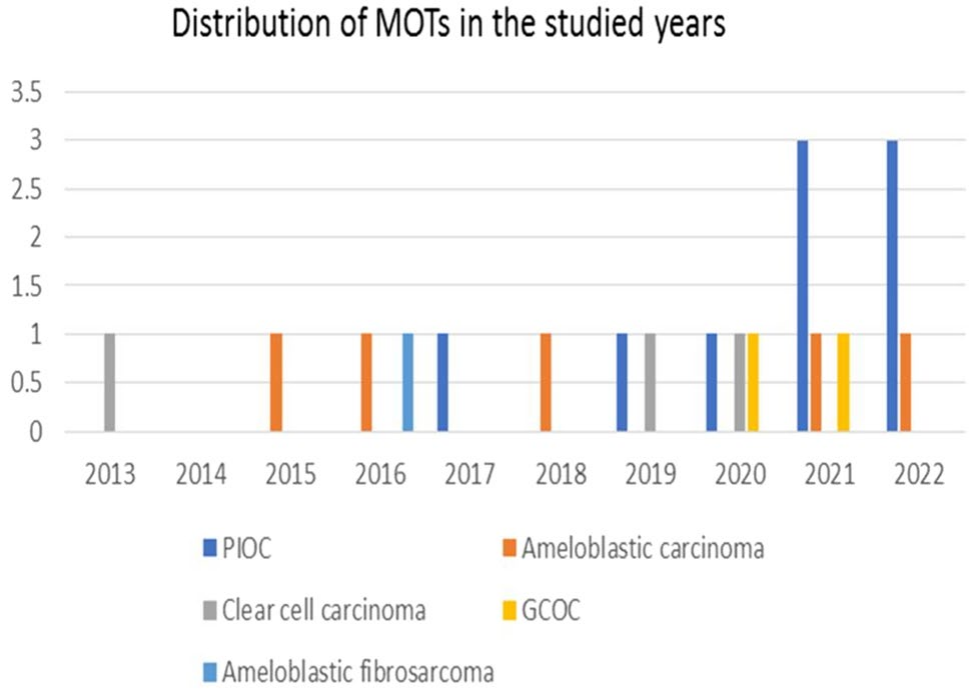
Distribution of malignant odontogenic tumors (MOT) between 2013 and 2022

Among the 5543 oral biopsies diagnosed in the Oral and Maxillofacial Pathology Department in the period between 2013 and 2022, 6.4% (n = 357) were diagnosed as OT. Within this percentage, 94.1% (n = 336) were benign, whereas 5.9% (n = 21) were MOT with a predominance of carcinomas, accounting for 95.2% (n = 20). The greatest incidence rate was in 2021 with 5 cases (25%), followed by 2022 with 4 cases (20%). No MOT was diagnosed in 2014 (Fig. 1).

Table 1 shows the number and percentage of each MOT type. The two most commonly occurring tumor types were primary intra-osseous carcinoma (PICO NOS) (n = 10, 47.6%) and ameloblastic carcinoma (AC) (n = 5, 23.8%), followed by clear cell odontogenic carcinoma (CCOC) (n = 3, 14.3%), ghost cell odontogenic carcinoma (GCOC) (n = 2, 9.5%) and ameloblastic fibrosarcoma (AFS) (n = 1, 4.7%).

**Table 1:** Characteristics of malignant odontogenic tumors (MOT) in the studied period 2013–2022

Type of MOTs	Number	Sex		Age groups							Site	
		M	F	10–19	20–29	30–39	40–49	50–59	60–69	70–79	Mandible	Maxilla
PIOC NOS	10 (47.6)	6	4	–	–	–	6	2	2	–	5	5
AC	5 (23.8)	5	0	–	1	1	2	–	1	–	3	2
CCOC	3 (14.2)	1	2	–	–	–	1	1	–	1	3	–
GCOC	2 (9.5)	0	2	1	–	1	–	–	–	–	–	2
AFS	1 (4.7)	0	1	–	–	1	–	–	–	–	–	1
Total	21	12	9	1	1	3	9	3	3	1	11	10
Percentage		57.1%	42.9%	4.7%	4.7%	14.3%	42.9%	14.3%	14.3%	4.7%	52.4%	47.6%

*PIOC NOS* primary intraosseous carcinoma, *AC* ameloblastic carcinoma, *CCOC* clear cell odontogenic carcinoma, *GCOC* ghost cell odontogenic carcinoma, *AFS* ameloblastic fibroscarcoma

### Frequency of MOT According to Patient Sex

Table 2 shows the number of MOT according to sex showing (57.1%) slight male predominance (1.3:1). PIOC NOS and AC occurred more frequently in male patients, whereas CCOC and GCOC occurred more frequently in female patients. The only diagnosed AFS occurred in a female patient.

**Table 2:** Frequency of malignant odontogenic tumors (MOT) by location

Type of MOT	Mandible			Maxilla		
	Right side(n)	Left side (n)	Crossing midline (n)	Right side (n)	Left side (n)	Crossing midline (n)
PIOC NOS	2	2	1	1	2	2
AC	3	–	–	1	1	–
CCOC	2	–	1	–	–	–
GCOC	–	–	–	–	2	–
AFS	–	–	–	–	1	–
Total	7	2	2	2	6	2

*n* number of cases, *PIOC NOS* primary intraosseous carcinoma, *AC* ameloblastic carcinoma, *CCOC* clear cell odontogenic carcinoma, *GCOC* ghost cellodontogenic carcinoma, *AFS* ameloblastic fibrosarcoma

### Frequency of MOT According to Patient Age

The reported frequency according to the age ranged from 10 to 79 years with the elevated incidence observed in the middle and old age groups of patients (n = 19, 90.4%) with a median age being 42. PIOC NOS was the most common tumor type diagnosed in 40s patients followed by 50s (Table 3).

**Table 3:** Characteristics of the recurrent malignant odontogenic tumors (MOT)

Recurrent lesion	Age	Sex	Site	Clinical presentations	Radiological features		Diagnosis of the Primary lesion	Time interval between 1ry & recurrent lesions	Deaths	Follow-up period
GCOC	44	M	Maxilla extending from the second premolar till maxillary tuberosity	Fast-growing swelling with buccal expansion and surface ulceration Negative in aspiration The related teeth except the upper second molar	Ill-defined radiolucent lesion causing severe bone expansion, thinning of the cortical plates of bone, and buccal perforation	DGCT		1 year	Null	Every 3 months in first year then every 6 months till now (2019–2024)
GCOC	30	F	Maxilla extending from the left lateral incisor till the first molar	Large recurrent swelling Negative in aspiration. All related teeth were vital Enlarged and palpable lymph nodes	An ill-defined radiolucent lesion with bone expansion and thinning of the cortical plates. Obvious perforation of the buccal cortical plate	Hybrid tumor (AOT & AB)		1.5 years	Null	Every 3months in the first year, then every 6 months till now (2020–2024)
PIOC NOS	41	M	Maxilla, extending from the right first premolar to the left canine	Large diffuse maxillary swelling crossing the midline with necrosis Few drops of pus and blood on aspiration. Related teeth were endodontically treated except the upper right first premolar	Ill-defined radiolucent lesion causing expansion and thinning of the cortical plate of bone obvious perforations in some areas	Undiagnosed primary tumor (it was reported to be a slowly growing cystic lesion related to non-vital anterior maxillary teeth)		N/A*	Null	Every 3 months in the first year, then every 6 months till now (2020–2024)

*GCOC* ghost cell odontogenic carcinoma, *PIOC NOS* primary intraosseous carcinoma, *DGCT* dentinogenic ghost cell tumor, *AOT* adenomatoid odontogenic tumor, *AB* ameloblastoma, *N/A* not available

### Frequency of MOT According to Site

A slight predilection of the mandibular cases recorded as 55% (n = 11) was noted in the comparison to the maxillary cases recorded as 45% (n = 9).

### Frequency of Recurrent MOT

Across all MOT (n = 21), 85.7% (n = 18) of cases appeared de novo while 14.3% (n = 3) were recurrent cases of OT. Subgroup analysis identified the average patient age at 38.2 years (range 30–44 years) with 66.66% male and 33.33% female. All recurrent MOT appeared in the maxilla. Two cases were diagnosed as GCOC while the last one was PIOC NOS.

**Fig. 2:**
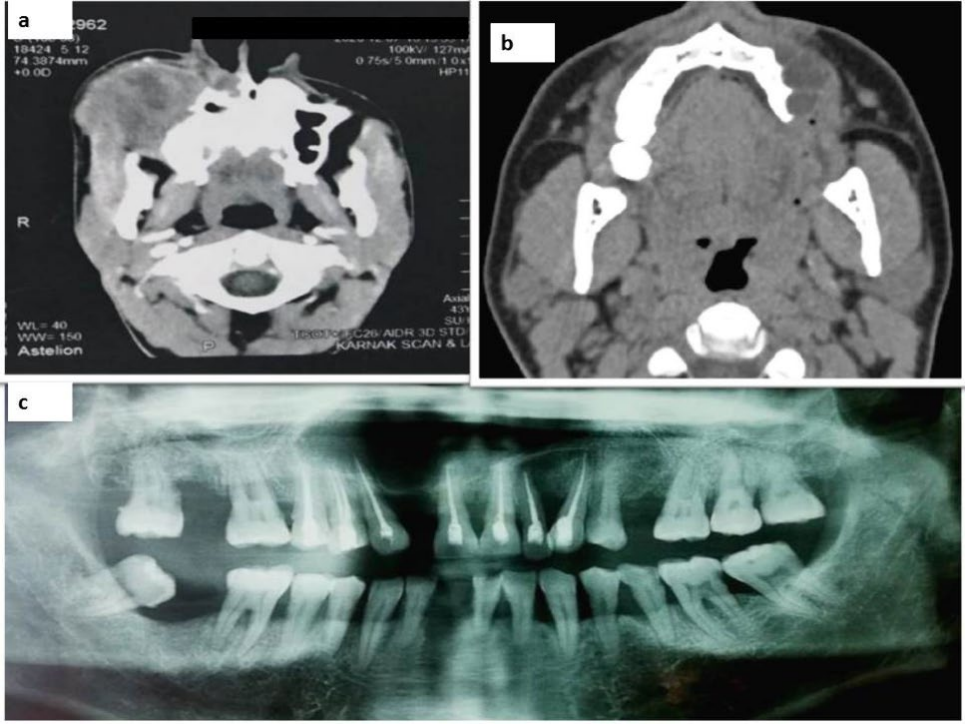
The radiographic presentation of the 3 recurrent MOT cases. **a** the first case was GCOC in the maxilla extending from the second premolar till maxillary tuberosity causing severe expansion and perforation of buccal bone, **b** the second case was GCOC in the maxilla extending from the left lateral incisor till the first molar causing obvious destruction of bone and perforation and, **c** the third case was PIOC NOS in the maxilla extending from the right first premolar to the left canine causing expansion and thinning of cortical plates of bone. *MOT* malignant odontogenic tumor, *GCOC* ghost cell carcinoma, *PIOC NOS* primary intraosseous carcinoma

**Fig. 3:**
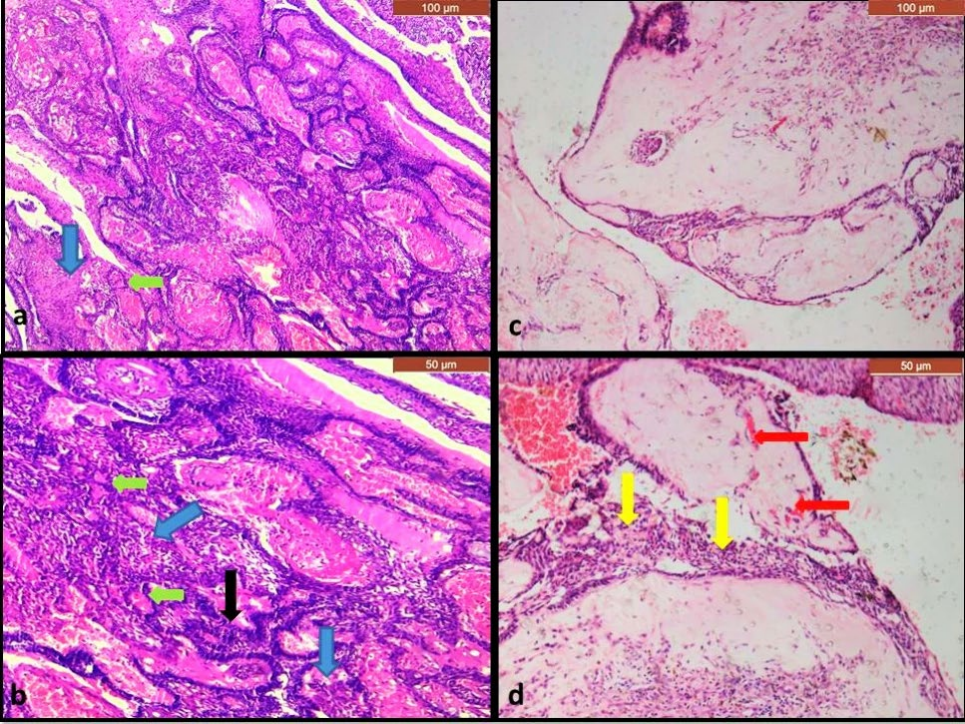
Photomicrographs of histological sections of primary benign OT, a case of hybrid odontogenic tumor showing areas of plexus of odontogenic epithelial cells (black arrow) as seen in AB, in addition to whorls of spindle cells (blue arrows) and some duct-like structures (green arrows) as seen in AOT (**a**; H&Ex100& **b**: H&E × 200), a case of DGCT showing odontogenic epithelial cells with some ghost cells (yellow arrows) and large areas of dentinoids (red arrows)(**c**; H&Ex100& **d**: H&E × 200). *OT* odontogenic tumor, *AB* ameloblastoma, *AOT* adenomatoid odontogenic tumor, *DGCT* dentinogenic ghost cell tumor

**Fig. 4:**
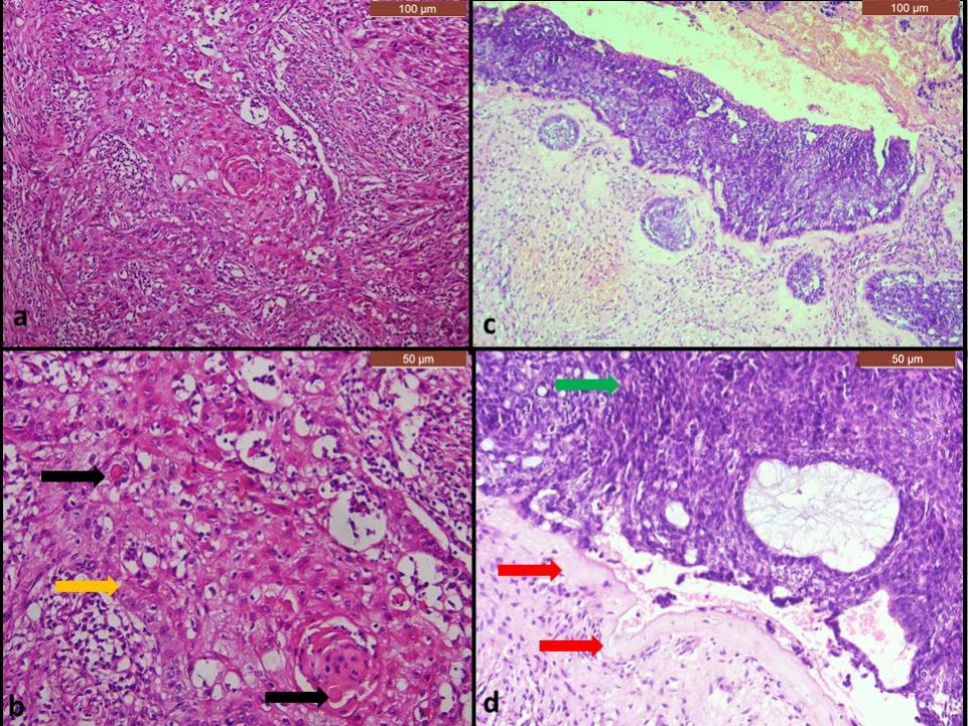
photomicrographs of histological sections of recurrent MOT, a case of PIOC NOS showing masses of discohesive malignant epithelial cells (yellow arrow) and cells showing single-cell keratinization (black arrows) (**a**; H&Ex100& **b**: H&E × 200), a case of GCOC showing odontogenic epithelial cells with prominent dysplastic features, some ghost cells (green arrow) and areas of dentinoids (red arrows) (**c**; H&E × 100& **d**: H&E × 200). *MOT* malignant odontogenic tumor, *PIOC NOS* primary intraosseous carcinoma, *GCOC* ghost cell carcinoma

**Fig. 5:**
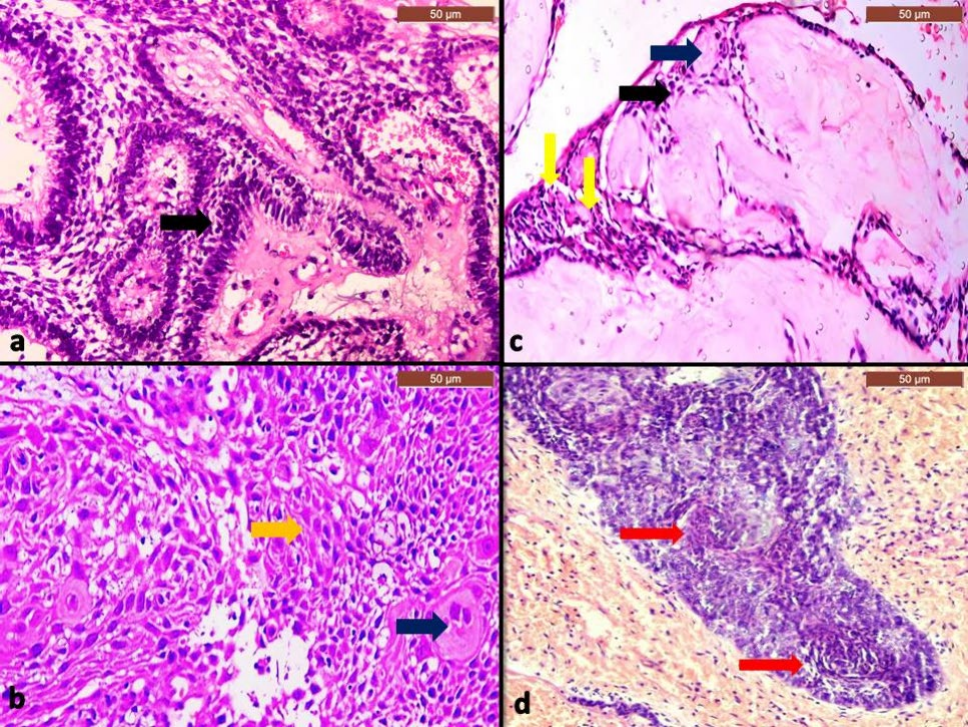
Higher magnified photomicrographs of histological sections of primary and recurrent MOT cases, **a** case of hybrid odontogenic tumor showing plexus of odontogenic epithelium (black arrow) surrounded by delicate connective tissue stroma, **b** PIOC NOS case showing malignant squamous epithelial cells (yellow arrow) and malignant multinucleated giant cell (blue arrow), **c** a case of DGCT showing odontogenic epithelial cells (black arrow) with scattered ghost cells (yellow arrow) and areas of dentinoids (blue arrow), **d** GCOC showing a mass of malignant odontogenic epithelium forming whorls pattern in some areas (red arrows) (a,b,c &d H&E × 200). *MOT* malignant odontogenic tumor, *OT* odontogenic tumor, *PIOC NOS* primary intraosseous carcinoma, *DGCT* dentinogenic ghost cell tumor, *GCOC* ghost cell carcinoma

After a rigorous examination of these cases, detailed information was reported with proper radiographic presentation as seen in Fig. 2. The first case was completely excised with safety margins and diagnosed as a hybrid odontogenic tumor composed of adenomatoid odontogenic tumor (AOT) and ameloblastoma (AB) (Figs. 3, 5) and recurred as GCOC (Figs. 4, 5). The second case was diagnosed as dentinogenic ghost cell tumor (DGCT), which was entirely removed by enucleation but it recurred after 18 months as GCOC (Figs. 3–5).

Regarding the third case, the available data on the primary lesion revealed that the patient had slowly growing swelling related to non-vital anterior maxillary teeth. The radiographic picture was described as a well-defined radiolucency with slight bony expansion. It was also reported that a clear straw-like fluid was found in the aspirate. The excisional biopsy was performed in a private clinic and the excised tissue was cystic on the cut section. The differential diagnosis of an odontogenic cyst was given but the lesion was not submitted for histological confirmation. When the lesion recurred, it was excised and diagnosed as PIOC NOS (Figs. 4, 5). The detailed description of the recurrent cases is summarized in Table 3. The treatment of these recurrent MOT cases was extensive surgical removal with safety margins with frequent and close follow-up visits (every 3 months) to minimize the chances of recurrence and metastasis. No recurrence was reported in the follow up reports of these patients till now. Most OT are well-known in the scientific literature for high recurrence rates, thus follow-up visits may be needed for years [5].

## Discussion

MOT are extremely rare intraoral entities that arise either de novo from elements of the tooth-forming apparatus or as a malignant transformation of a pre-existing oral lesion such as a benign OT or cyst [6]. In the latest updates of the Head and Neck WHO classification, our understanding of the evolution of MOT is expanded [5].

According to the current literature, the frequency of development of malignancy in recurrent benign OT has not been well studied. Therefore, we aimed to investigate the prevalence of MOT and to report the relative rate of malignant transformation in recurrent benign OT.

Generally, OT comprised a quite small fraction of intra-oral lesions. In this study, we found that OT accounted for only 6.4% of the cases in which oral biopsies had been performed. The majority of OT was benign accounting for 94.1% of the studied cases. The frequency of MOT accounted for only 5.8% which is consistent with the studies conducted in Brazil (5.5%) [7], Turkey (5.5%) [8], China (6.1%) [9], as well as the United Kingdom (5.7%) [10]. However, a lower fraction was stated in studies conducted in Malaysia (1.2%) [11]. On the other hand, a higher frequency was observed to be nearly 8.9% of all oral tumors in Africa and Asia [12, 13].

In our study, we found that benign tumors were the most frequent among all cases of OT (benign 94.1%, malignant 5.9%). MOT are rare, and only 21 (5.9%) patients with such tumors were recognized in this study similar to a previously performed study [14]. The most common MOT was detected in this study to be PIOC NOS, which was in agreement with the previously reported studies [1, 10], followed by AC, CCOC, and GCOC. Our noted finding was unlike most of the earlier performed studies that showed AC was the most frequently reported MOT. In addition our finding was consistent with the case series described in 2010 by Gupta and Ponniah [15].

The least common histological diagnosis was documented to be AFS, a subtype of odontogenic sarcoma, as defined in the most recent WHO classification [5]. Over the last 10 years, a single case of odontogenic sarcomas was observed but without any reported case of odontogenic carcinosarcomas, emphasizing the relative scarcity of this type of OT compared to carcinomas of odontogenic origin [5]. The reason for the absence of carcinosarcoma in this current study and many others can be attributed to being an unrecognized entity until the updated classification of WHO of head and neck tumors in 2017 [16].

The observed differences in the distribution of MOT might be due to the geographical and cultural variation among different study populations. Among the MOT, the incidence of odontogenic carcinoma is much higher than sarcomas accounting for 95.2% of cases which is in accordance with other studies [16]. Generally, the current study documented a recent increase in the frequency of MOT in Egypt. Our reported finding may be due to socioeconomic and cultural standards that obscure both the incidence and reporting of tumor types. Decreased reporting and delayed medical attention, of course, would affect the rate of recurrence and the ability to report neoplastic malignant transformation in such cases.

Although MOT may be found in any age group, their highest incidence was reported in patients in at least the fifth decade with more than 75% of cases occurring in those aged ≥ 40 years [2, 16, 17]. In this study, the overall male-to-female ratio among patients with MOTs was approximately 1.5:1. The male predominance detected in this study agrees with other published studies [18, 19]. Conversely, few studies have stated equal proportions of patients of both genders [20].

Across all the MOT, 85.7% (n = 18) were de novo while only 14.3% (n = 3) developed recurrence. Our study showed a marked maxillary involvement of recurrent MOT. All these tumors occurred as intra-osseous jaw lesions. The average patient age of recurrent MOT was 38.4 years with a slight male predilection (66.6%).

Among the recurrent MOT, two cases were diagnosed as GCOC. GCOC is a rare MOT and is considered to arise either de novo or from a calcifying odontogenic cyst (COC) or (DGCT) [21]. The first case in our study was GCOC which recurred from DGCT after its malignant transformation. Such a finding was documented previously from studies that highlighted that the development of GCOC from DGCT might take several years [22, 23].

The second case was developed from a previously diagnosed hybrid odontogenic tumor formed of AB and AOT. Hybrid odontogenic tumor types have been first documented by Yamazaki et al. in 2014, who stated that their occurrences are rare [24]. To the best of our knowledge, this is the second documentation of GCOC arising in a previously diagnosed hybrid tumor of AB and AOT [25].

GCOC was twice as often reported in the maxilla with a predilection to males in their fourth decade. Patients typically present with signs of a rapid painful swelling and mucosal ulcer which is in accordance with our study [26–28]. Findings related to radiographic examination were confirmed to be a powerful tool during the malignant transformation of such cases. Those cases were more likely to present as expansile lesions with ill-defined margins, causing destruction and perforation of the cortex of jaw bones similar to our documented cases [28 & 29].

We reported only one recurrent case of PIOC NOS from a long-standing undiagnosed well-defined cystic lesion in the anterior maxillary tooth-bearing area. PIOC NOS of the jaw is a rare neoplasm that can arise either from the lining epithelium of long-lasting odontogenic cysts or de novo from odontogenic epithelial rests [29, 30]. The commonly associated cysts with PIOC NOS are odontogenic keratocysts, residual, and dentigerous cysts [31–33]. Unfortunately, in our study, the initial occurrence of the lesion in our case was undiagnosed.

The peak incidence of PIOC NOS is in the sixth decade of life; however, it has a wide age range from 14 to 80 years [5]. A male predilection with a ratio (2:1) has been witnessed by many authors similar to our study [5, 33]. The lower jaw was more clinically affected (especially the posterior region) than the upper jaw [32, 33]. In the current study, the incidence in an unusual location in the anterior maxilla extending from the left canine area to the right first premolar area crossing the midline was detected. Many authors accredited that the chronic inflammatory process is the main prompting factor for PIOC NOS development [34, 35]. This may explain the presence of a purulent aspirate and necrosis caused by tumorigenesis.

Radiographic imaging showed an ill-defined radiolucent lesion with cortical bone thinning and perforation simulating another previous case series [34, 35]. Clinically, the early stage of PIOC NOS might frequently be asymptomatic or might cause mild dental disorders. Therefore, lymph node metastasis may already be present during the biopsy, which was an obvious finding in our case [36]. In the end, one limitation of this study is that the included cases are from one institute; this was attributed to the lack of a standard patient filing system in many governmental hospitals in addition to the lack of patient awareness of the importance of follow-up visits.

## Conclusion

Subtypes of OT vary according to genetic and/or environmental (epigenetic) factors. Any primary or recurrent lesions showing rapid growth and ill-defined borders radiographically should be biopsied in a timely fashion. Following the excision of any OT, clinical follow-up is essential, especially in older patients, given the risk of recurrence or malignant transformation.

## Data Availability

The datasets generated during the current study are not publicly available [patients’ data was kept confidential] but are available from the corresponding author upon reasonable request.
